# Trochleoplasty for patellofemoral instability: current concepts

**DOI:** 10.51894/001c.168165

**Published:** 2026-08-28

**Authors:** Srikanth Mudiganty, Aaron Hess, Mathew Nguyen, Joseph Gonzales, Rutledge Carter Clement, Dominic Gargiulo

**Affiliations:** 1 Orthopedics Community Health Systems - AllianceHealth Durant; 2 Orthopedics Michigan State University; 3 Orthopedics Louisiana State University Health Sciences Center New Orleans

**Keywords:** Patellofemoral instability, Trochleoplasty, Trochlea dysplasia, outcomes, complications.

## Abstract

**PURPOSE:**

Trochlear dysplasia is recognized as a key anatomical and biomechanical contributor to recurrent patellar instability. Trochleoplasty has re-emerged in contemporary practice as a surgical option specifically for patients with severe dysplasia leading to recurrent episodes of instability. This clinical review aims to summarise the latest evidence regarding the indications, types, surgical techniques, and clinical outcomes associated with trochleoplasty.

**RECENT FINDINGS:**

Trochleoplasty can be broadly categorized into three main types: lateral facet elevation, recession wedge, and sulcus-deepening procedures. Among sulcus-deepening methods, the modified Bereiter technique—often referred to as the “thin flap” approach—has gained popularity in recent years over the traditional Dejour “thick flap” technique. A more recent development is the arthroscopic variant of the Bereiter procedure, offering a minimally invasive alternative. Contrary to previous reports, recent literature demonstrates notable improvements in patient-reported outcomes, patellofemoral stability, and overall satisfaction following these procedures.

**SUMMARY:**

Sulcus-deepening trochleoplasty is an established surgical option for managing patients with high-grade trochlear dysplasia associated with patellofemoral instability. It has been linked to reduced recurrence rates, low complication risks, and favourable clinical outcomes. Orthopaedic surgeons treating patellofemoral disorders should be familiar with the appropriate indications and techniques for this procedure.

## INTRODUCTION

Trochlear dysplasia is an abnormality where the trochlea is shallow, flat, or convex, often with a superolateral spur .^1^ The dysplasia provides an inconsistent mediolateral constraint for patellofemoral articulation leading to instability.^2^ It has been described as the most significant anatomic risk factor for patellar instability in all age groups.^3–5^ High grade trochlear dysplasia is associated with altered kinematics in the coronal plane leading to lateral patella tilt and patellofemoral impingement in the sagittal plane, which may increase the risk of patellofemoral osteoarthritis.^6,7^ Studies have reported less than 2% incidence of trochlear dysplasia in normal population in contrast to up to 96% in cases of patella instability.^8,9^

Trochleoplasty is a procedure to improve the femoral trochlea groove morphology and restore congruency. It involves excising the supratrochlear spur and generates a new anatomically aligned groove position. The goal of trochleoplasty is to enhance patellofemoral stability and prevent patellar dislocation. Trochleoplasty is usually combined with other surgical interventions namely Tibial tuberosity osteotomy, lateral release and most commonly with medial patellofemoral ligament reconstruction (MPFL).^10^

This clinical review highlights the features of trochlea dysplasia and discusses the use of trochleoplasty to treat patients with patellofemoral instability. We review the different trochleoplasty techniques that have been described and present the published clinical outcomes.

### Classification

A single valid, comprehensive, and reproducible classification for trochlea dysplasia has not yet been described. The Dejour classification of trochlea dysplasia is the most frequently used classification in literature.^1^ It is based on lateral and axial imaging of the distal femur. Dejour identified trochlea flattening based on the where the trochlea floor crosses anterior to the lateral condyle on 30-degree knee flexion lateral x-ray (crossing sign).^8^ Four different types of trochlea dysplasia A-D were then described.^11^ (Table 1 - Radiographic findings by Dejour dysplasia type).^12^

**Fig. 1.**
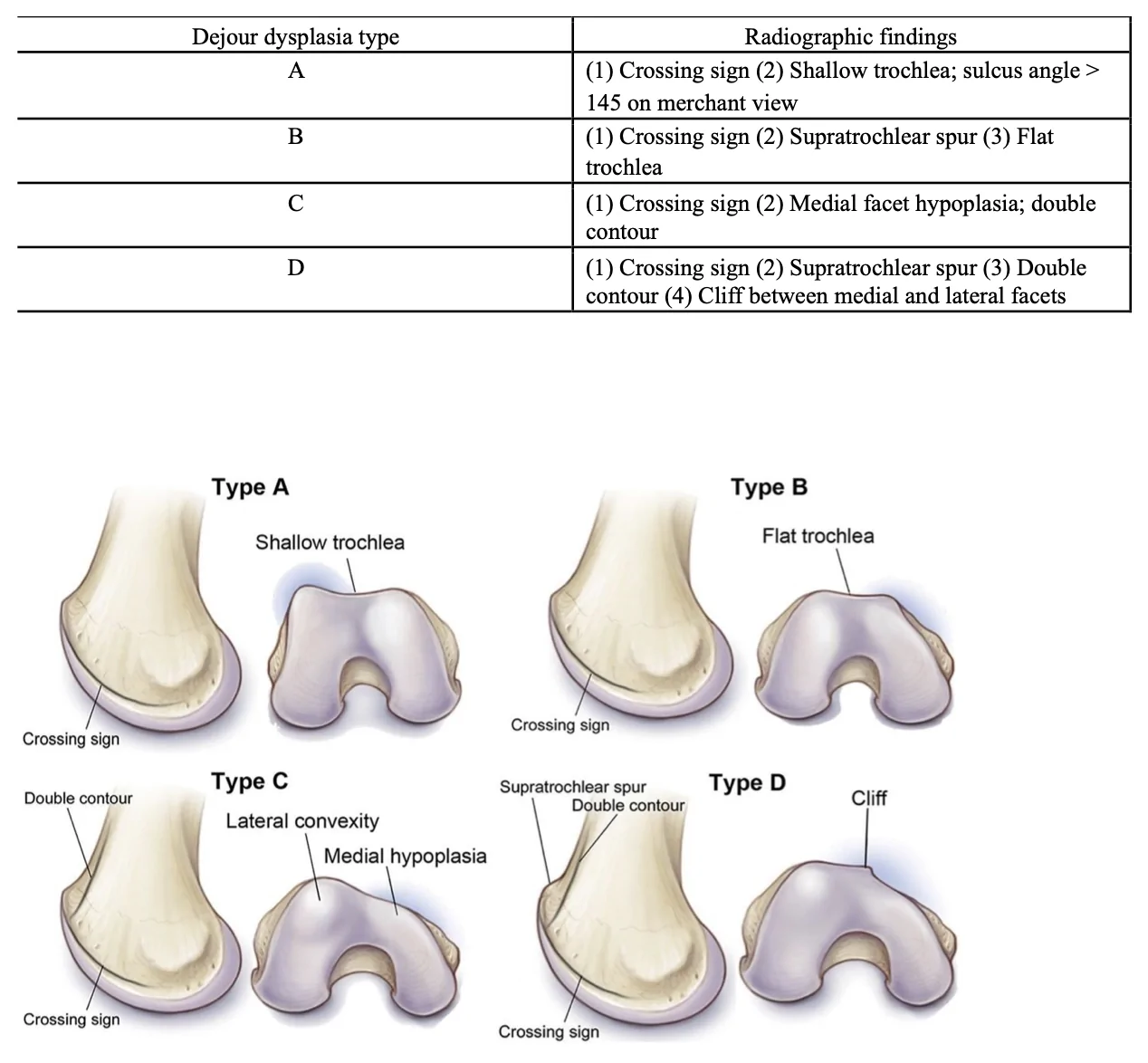
Dejour classification Dejour classifications of trochlear dysplasia. Type A: Crossing sign, trochlear morphology preserved (fairly shallow trochlea, >145°). Type B: Crossing sign, supratrochlear spur, flat or convex trochlea. Type C: Crossing sign, double contour (projection on the lateral view of the hypoplastic medial facet). Type D: Crossing sign, supratrochlear spur, double contour, asymmetry of trochlear facets, vertical link between medial and lateral facet (cliff pattern). [Used and adapted from^13^https://creativecommons.org/licenses/by-nc-nd/4.0/]

While the Dejour classification has gained broad acceptance in the literature, it has poor reproducibility.^14^ Dichotomous classification of trochlea dysplasia into high and low grades have more reliability and have been described in recent literature.^12^

Magnetic resonance imaging (MRI) has been utilized to understand the morphology of trochlea dysplasia. It helps to evaluate the cartilaginous sulcus which does not follow the bony anatomy in normal patients^15^ but matches more commonly in flat or convex trochlea.^1^ The Oswestry-Bristol classification (OBC) is an MRI-based classification described in 2020 by Sharma et. Al.^16^ This is a 4-grade classification for trochlear dysplasia namely - A - Normal, B - Mild (shallow trochlea), C - Moderate (Flat trochlea), D - Severe (Convex trochlea). It has been validated and is more reproducible and reliable^17^ compared to the Dejour classification.^5^ OBC is advantageous due to its simplicity and ability to categorize trochlear dysplasia relevantly with standard T2 weighted knee MRI images.^18^

**Fig. 2.**
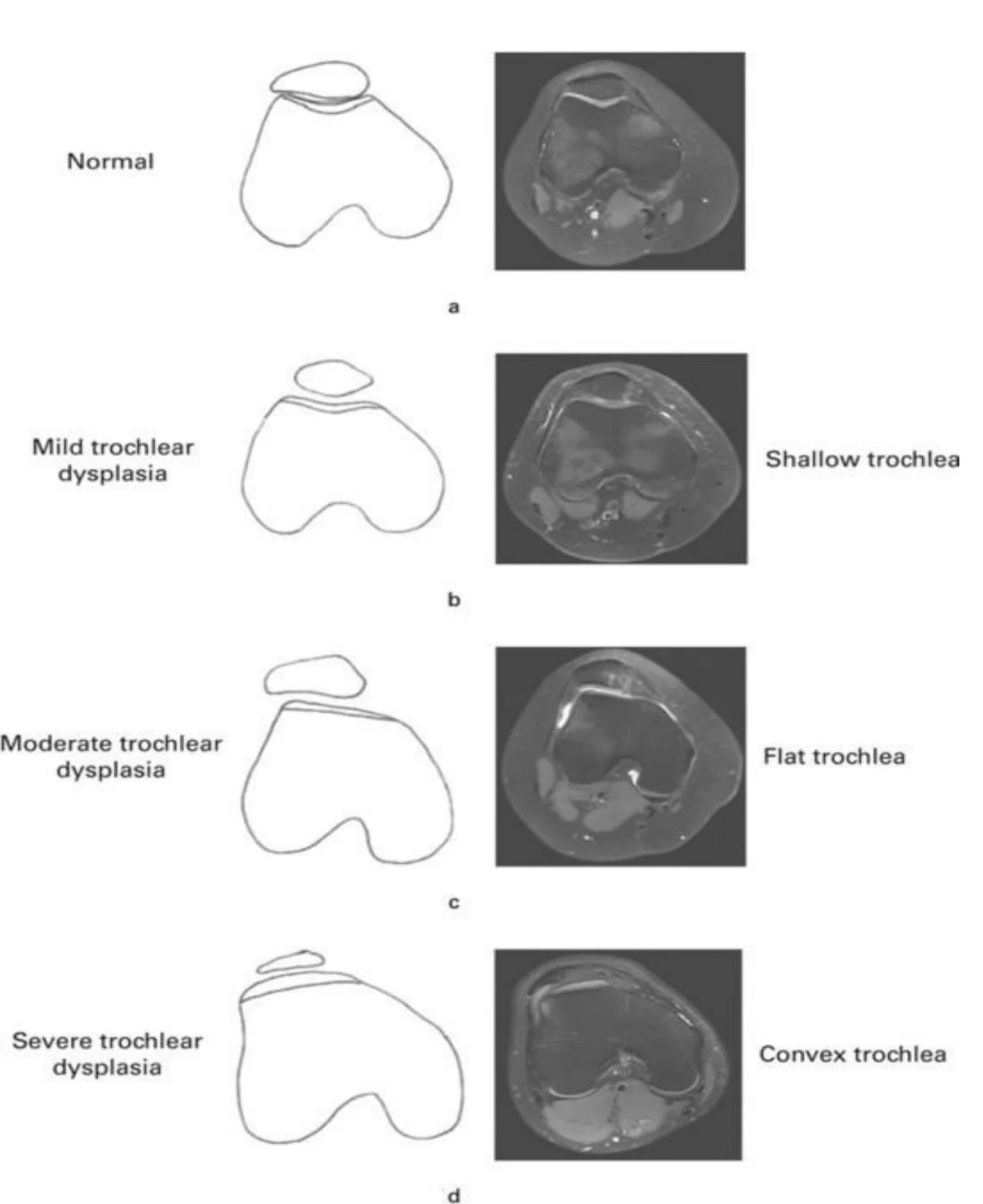
OBC classification [Used and adapted from^18^ https://creativecommons.org/licenses/by-nc-nd/4.0/] Classification of dysplastic trochlea does have major challenges. Tscholl et al.^19^ highlighted that Xray and MRI-based classification of trochlea dysplasia have only a fair reliability. Lateral Trochlea Inclination (LTI) is angle measured on axial MRI between the posterior condyle line and lateral trochlea facet.

**Fig. 3.**
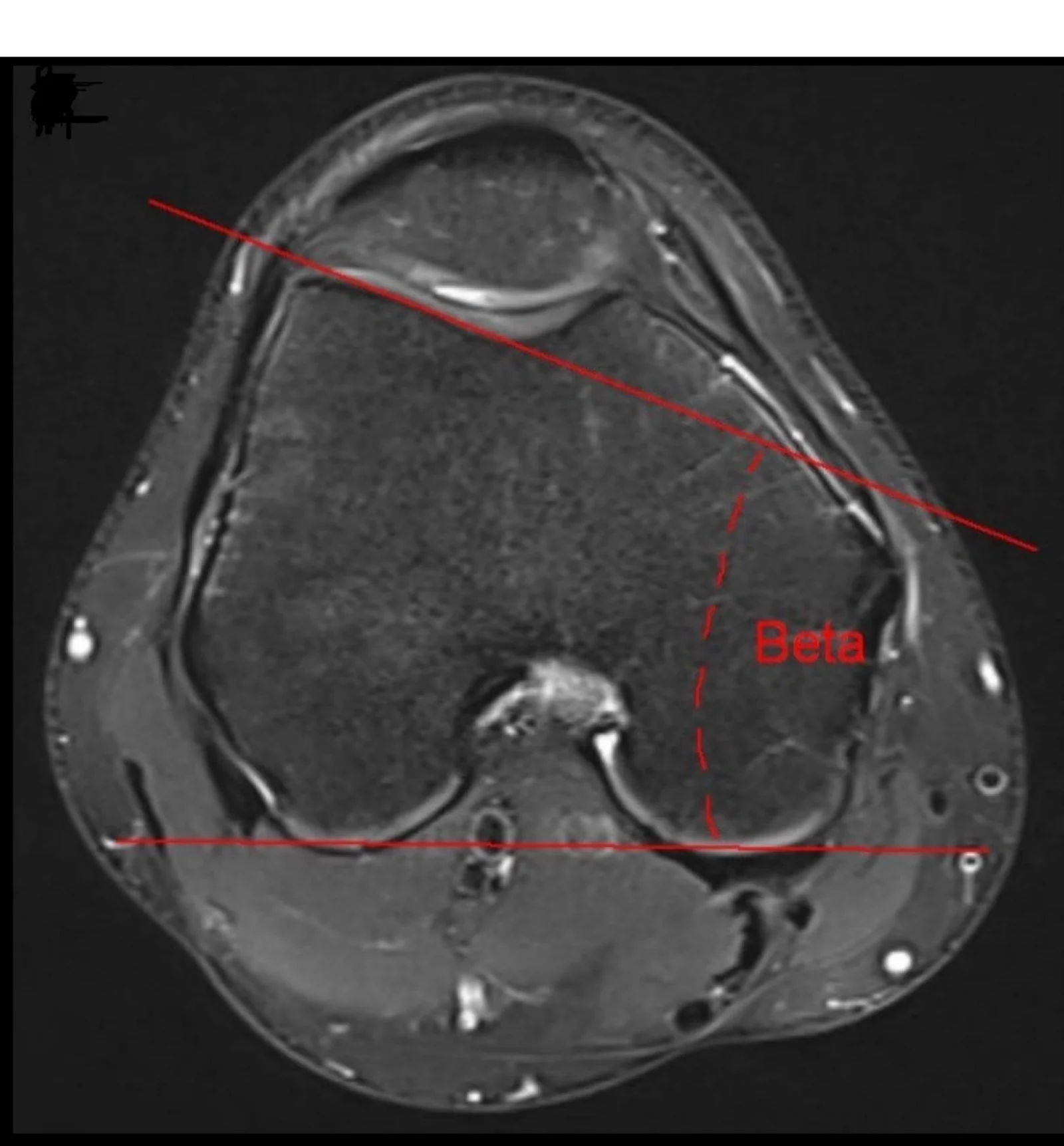
LTI single image Measurement of the lateral trochlear inclination. The lateral trochlear inclination is measured through an angle (beta) between two lines. The first line is connected to the posterior aspect of both femur condyles. The second line is placed on the lateral facet of the trochlea. [Used and adapted with changes to the figure legend from^20^ https://creativecommons.org/licenses/by/4.0/]

LTI has been described as the single most important objective measurement of trochlea dysplasia.^21^ According to Cheng et al, the normal mean is 21 and an LTI angle <9 indicates Trochlear Dysplasia.^22^ The newer 2-image MRI technique from Joseph et al,^21^ compared to single-image technique described by Carrillon et al.,^23^ the threshold for TD was reduced from 11 to 8.9.

**Fig. 4.**
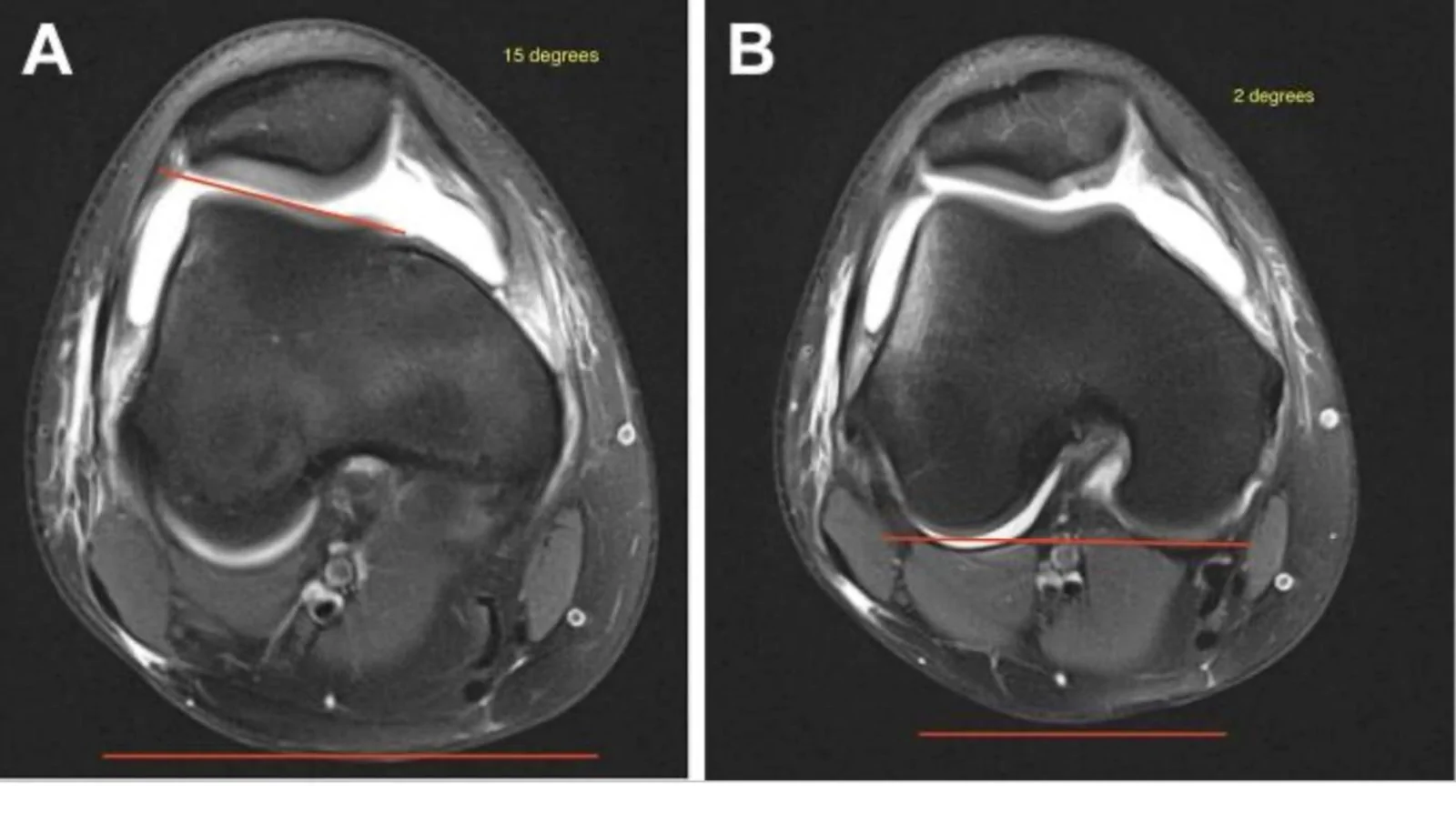
LTI double image [Used and adapted with changes to the figure legend from^21^ https://creativecommons.org/licenses/by-nc-nd/4.0/]

Measurement technique for 2-image lateral trochlear inclination (LTI). (a) An angular measurement was taken on an axial MRI image between the most proximal aspect of the lateral trochlear cartilaginous surface and a horizontal reference line represented by the red lines. This was the same image used for the single- image LTI. This angle measured 15°. (b) An angle was measured between the posterior condyles and a horizontal line represented by the red lines. This angle measured 2°. The 2-image LTI was determined by subtracting the angle of the posterior femoral condyles relative to the horizontal from the angle of the proximal lateral trochlea relative to the horizontal. In this example, the LTI calculation was 15° – 2° = 13°. This was 17° different from the single-image LTI.

### Indications and Contraindications

Trochleoplasty is ideally indicated in a patient with symptomatic recurrent patellar instability, severe trochlea dysplasia, absence of any patellofemoral joint degenerative changes and failure of conservative management. The role of trochleoplasty in primary management of recurrent patellar instability has been questioned. However, it has definitely been utilized in revision surgery of patients with failed attempts to treat patellar instability, where trochlea dysplasia was a significant unaddressed factor.^24^

Radiological evidence of high-grade trochlear dysplasia in presence of recurrent patellar instability validates performing trochleoplasty.^1^ Trochlear dysplasia types B and D are the indications of choice for sulcus deepening trochleoplasty when the supratrochlear spur is ≥ 5 mm.^7^ Type A trochlea dysplasia does not warrant any trochlea surgery.

Open physis has long been considered as an absolute contraindication for trochleoplasty due to physeal damage and subsequent growth disturbance. However, Nelitz et al.^25^ have reported favorable results of thin flap trochleopasty in a case series of 18 skeletally immature patients at average 2.3 years follow up. This is the first study to report the successful outcome of this procedure with no sequelae of physeal damage in adolescents with less than 2 years of growth remaining. However, patients with more than 2 years of growth remaining should not undergo this procedure due to risk of physeal injury. In contrast, trochleoplasty is technically more challenging in patients over the age of 30 due to sclerotic bone and less cartilage malleability.^26^

The contraindications for trochleoplasty can be absolute and relative. The absolute contraindications include permanent patellar dislocation, patellofemoral arthritis and Dejour Type A dysplasia. Preoperative pain is not an absolute contraindication and may occasionally improve after trochleoplasty.^27^

### Trochleoplasty techniques

Four basic Different trochleoplasty techniques have been outlined:

1. Lateral wedge augmentation trochleoplasty (LWAT) initially proposed by Albee^28^
2. Recession or recession-wedge trochleoplasty^29^
3. Sulcus deepening trochleoplasty, described by Masse, subsequently updated by Dejour^5^
4. Bereiter or thin flap trochleoplasty^12^

Patellofemoral congruence can be enhanced by raising the lateral facet of the trochlea but is associated with poor clinical results attributed to pain.

**Fig. 5.**
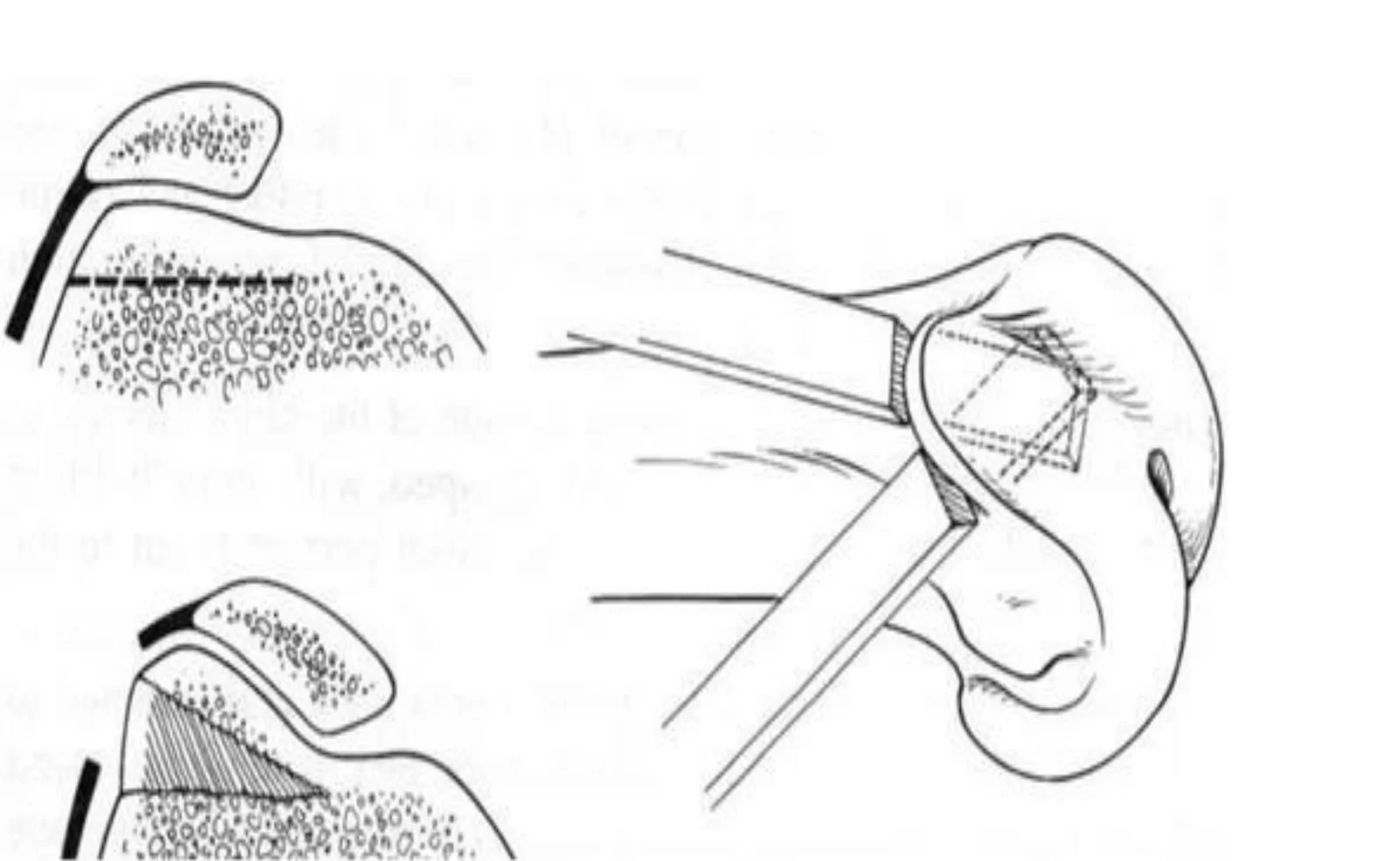
LWAT. Lateral Wedge Augmentation trochleoplasty. [Used and adapted with changes to the figure legend from^30^ http://creativecommons.org/licenses/2.0]

Recession wedge trochleoplasty was first described by Goutallier in 2002 and also published by Thaunat. It involves recession of pre-planned wedge of trochlea bone bump to create a deepened trochlea.^17^

**Fig. 6.**
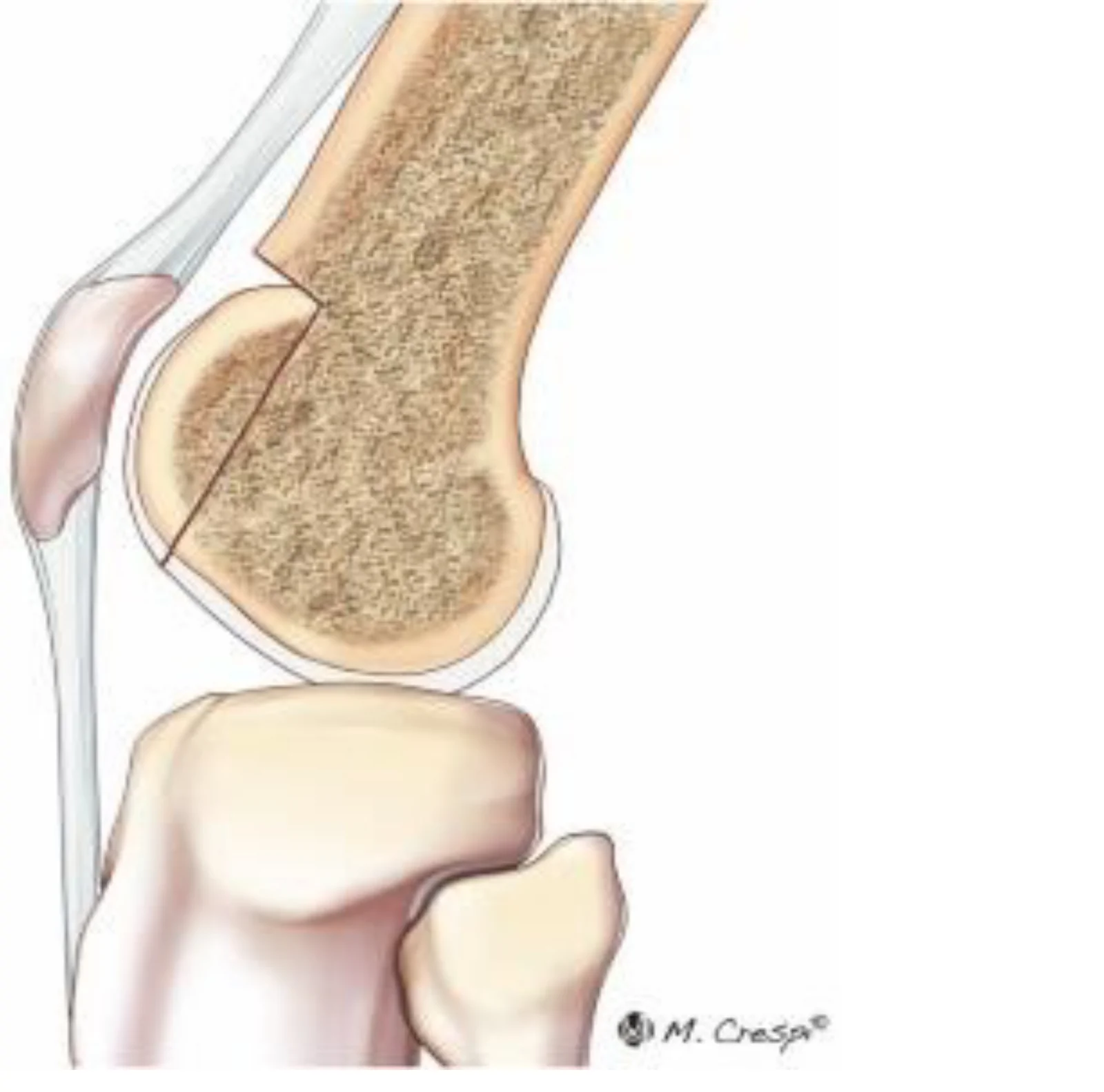
Recession wedge trochleoplasty (Goutallier) (Used and adapted with permission from^10^)

Deepening of the trochlea sulcus was first described by Masse by impacting the cartilage into the subchondral bone with no fixation. Dejour used the same principle to create a V-shaped trochlea groove by osteotomy of both femoral condyles.^5^

**Fig. 7.**
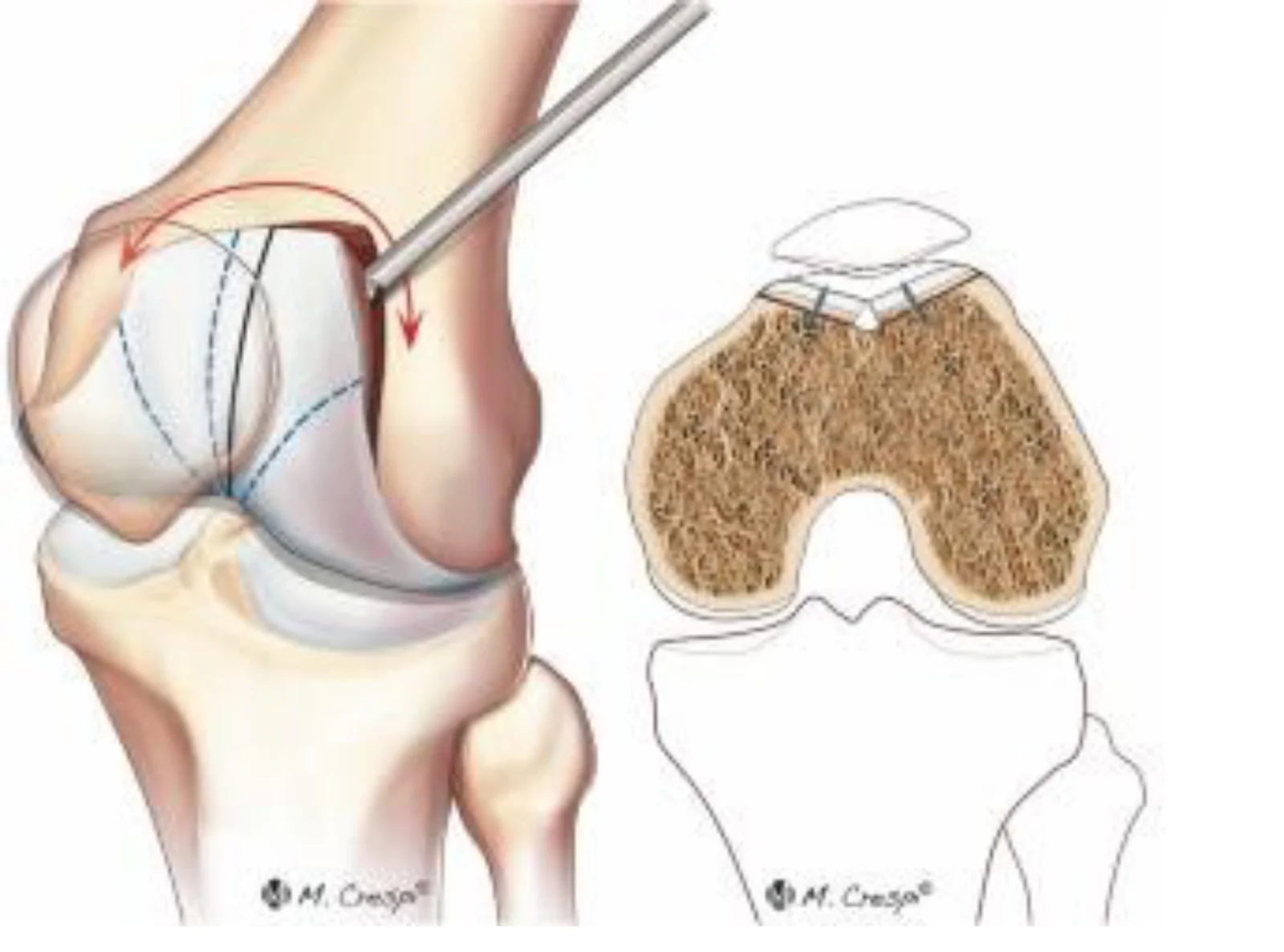
Deepening sulcus trochleoplasty (Dejour) (Used and adapted with permission from^10^)

Although this technique restores the proximal trochlea groove, it fails to address the trochlea up to the intercondylar notch. The thin flap or Bereiter technique as detailed by Von knoch et. al^12^ attempts to restore the normal trochlea anatomy by raising a trochlea osteochondral flap and fashioning a bony sulcus utilizing high speed burrs. The raised flap is moulded onto the underlying newly created sulcus and secured using a suture tape

**Fig. 8.**
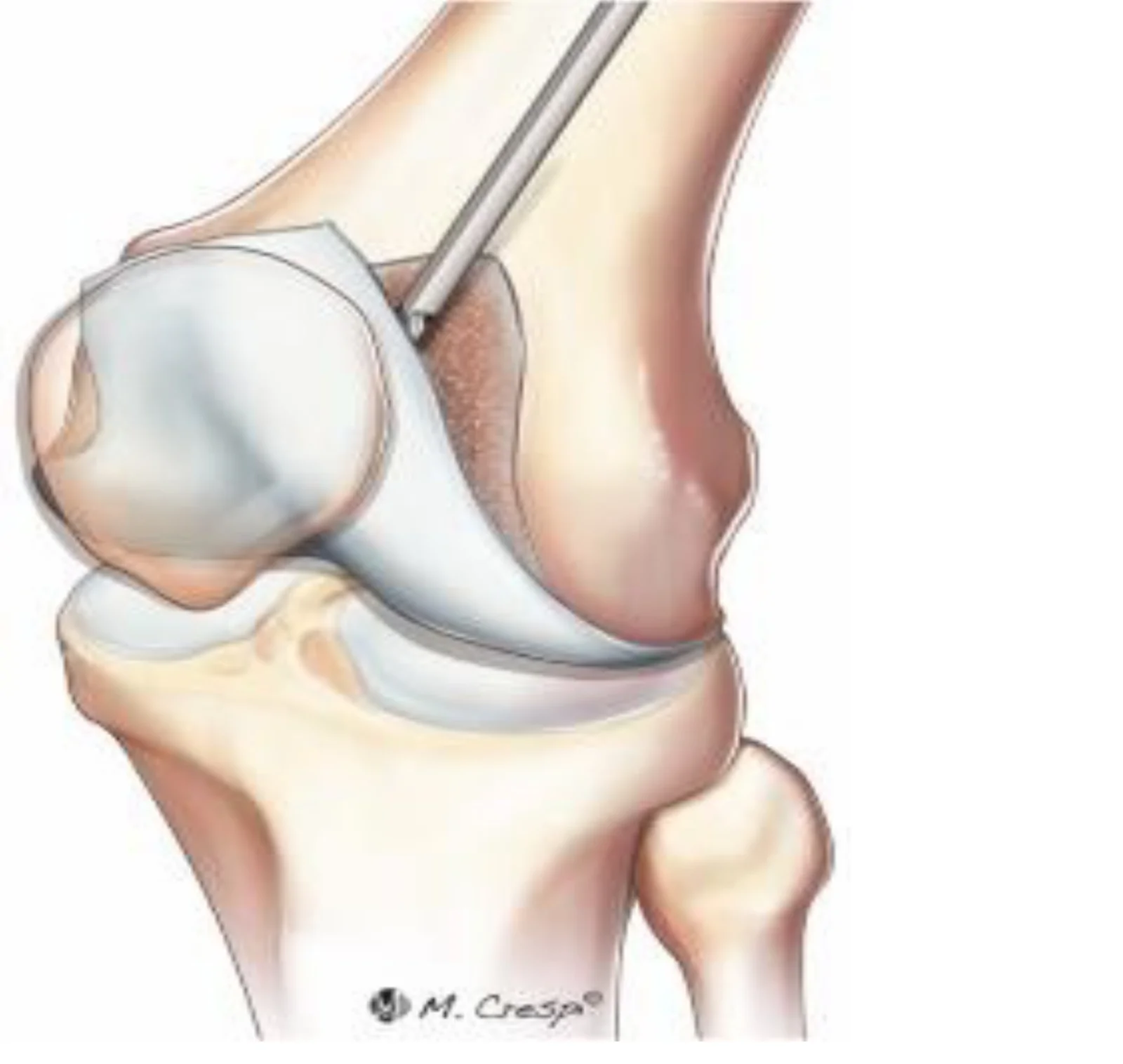
Thin flap trochleoplasty (Bereiter). (Used and adapted with permission from^10^)

This technique has the potential advantage of reproducing the normal trochlea anatomy without any uneven surfaces along the trochlea groove. The concave trochlear modelling has been visualised on post operative CT scans.^17^ Schöttle et al.^31^ examined the viability of the reattached osteochondral flap following trochleoplasty. Histological analysis revealed a normal cartilage matrix and cell distribution, along with a typical lamellar structure of the subchondral bone and evidence of successful flap healing.

Various modifications of the thin flap Bereiter technique have been published in literature. An arthroscopic variant, Arthroscopic Deepening trochleoplasty, has been detailed by Blond and Schottle in 2010.^32^ Lateral condyle lengthening combined with the thin flap Bereiter technique has been published.^33^ The harvested bone block is positioned beneath the osteochondral flap at the proximal aspect of the lateral femoral condyle. This provides lateral stabilization on full extension of the knee.

Precise Arthroscopic mini-trochleoplasty (PAM) as described by Xu et. al. in 2020^9^ is an all-arthroscopic technique. Its advantages include minimal invasion leading to less post-operative scarring, pain, and stiffness. Additionally, it also avoids the need for a medializing TTO is mildly elevated TT-TG, as it lateralizes the trochlear groove by at least 5 mm.

### Combined procedures

Instability of the patella cannot only to attributed to presence of an abnormal trochlea, which can be corrected with sulcus deepening trochleoplasty but also to other associated anatomical factors (eg. patella alta, abnormal TT-TG distance) and therefore, its management invariably involves combined procedures like MPFL reconstruction and medialization/distalisation of tibial tubercle.^26^ Thus, trochleoplasty is infrequently carried out as a standalone procedure.^6^ The TT-TG distance is increased in 56% of cases of patellofemoral instability compared to only 3.5% of normal knees.^34^ Therefore, medialization of the tibial tubercle is indicated in addressing patellar instability.^4^ Trochleoplasty itself leads to lateralization of the sulcus, negating the need for tibia tubercle medialization^28^ while some authors believe tibial tubercle transfer (TTT) provides better control over definite patella tracking.^35^

## METHODS

This manuscript is a clinical narrative review with a structured literature search.

The manuscript was prepared with consideration of the Scale of the Assessment of Narrative Review Articles (SANRA), including explicit aims, a description of the literature search, appropriate referencing, and presentation of relevant evidence.

### Eligibility criteria

All clinical studies describing various trochleoplasty techniques for management of patellofemoral instability were considered for eligibility. Articles published in English literature only were included. Only articles indexed in the PubMed database were included in this study. Reviews, editorials, opinion papers, or letters were excluded, as were studies involving animal models, in vitro experiments, cadaveric specimens, computational simulations, or biomechanical analyses. Furthermore, studies lacking quantitative outcome data were excluded from the final analysis.

### Search strategy

A comprehensive electronic literature search was conducted in January 2025 using PubMed for studies over a 25-year period from January 2000 to December 2024 with no minimum bar over number of patients or follow-up duration. The medical subject headings (MeSH) used as strings for PubMed search included (“trochleoplasty”[Title/Abstract] OR “sulcus deepening”[Title/Abstract]) AND (“patellar instability”[Title/Abstract] OR “trochlear dysplasia”[Title/Abstract]) AND (outcomes[Title/Abstract] OR complications[Title/Abstract] OR recurrence[Title/Abstract).

### Selection and data collection

Two authors (A.H. and M.N.) conducted an independent search across the selected databases. Titles were manually screened for thematic relevance, followed by a thorough evaluation of abstracts from potentially eligible publications. When inclusion appeared likely, full texts were obtained and assessed accordingly. The reference lists of all included full-text articles were also reviewed to capture any additional studies not identified during the initial search. Discrepancies between the reviewers were resolved through discussion and, if necessary, adjudicated by a third senior author (S.M.).

### Data items

Two reviewers (A.H. and M.N.) independently performed data extraction. The following data were systematically retrieved: first author and year of publication, journal name, study design, follow-up duration, number of included patients/Knees, mean age, sex distribution, type of Trochleoplasty, associated procedures, follow-up in years, clinical outcomes, complications and success rate.

### Study selection

The literature search resulted in 77 articles concerning the topic of interest. All search results were extracted and checked for relevance. Of these, 13 were discarded because they were duplicates. Following the defined inclusion criteria, abstracts of 64 articles were reviewed, and 28 studies were excluded because they did not fulfil the eligibility criteria. An additional nine articles were excluded because they did not offer quantitative data on the outcomes of interest. In conclusion, 27 investigations were included in the present analysis. Of them, 26 were Case reports or series, and one had a prospective design.

## DISCUSSION

In this manuscript, we included all case series’ published over the last 25 years (January 2000 to December 2024) in PubMed electronic database with no minimum bar over number of patients or follow up duration. The rationale of this criteria was to include all relevant studies highlighting various trochleoplasty techniques including demographic features, associated procedures, outcome scores and complication rates. The most common additional procedure performed with a trochleoplasty was a medial patellofemoral ligament reconstruction (MPFLr). The follow-up duration in studies included in this manuscript ranged from 1-31 years. These studies demonstrate a statistically significant improvement in patient-reported outcome measures (PROMs) following trochleoplasty with Kujala score and International Knee Documentation Committee (IKDC) score as the most frequently reported outcome measures. Successful outcomes were noted in majority of the patients (60-100%) with minimal complication rates (0-28%).

**Table 2.**
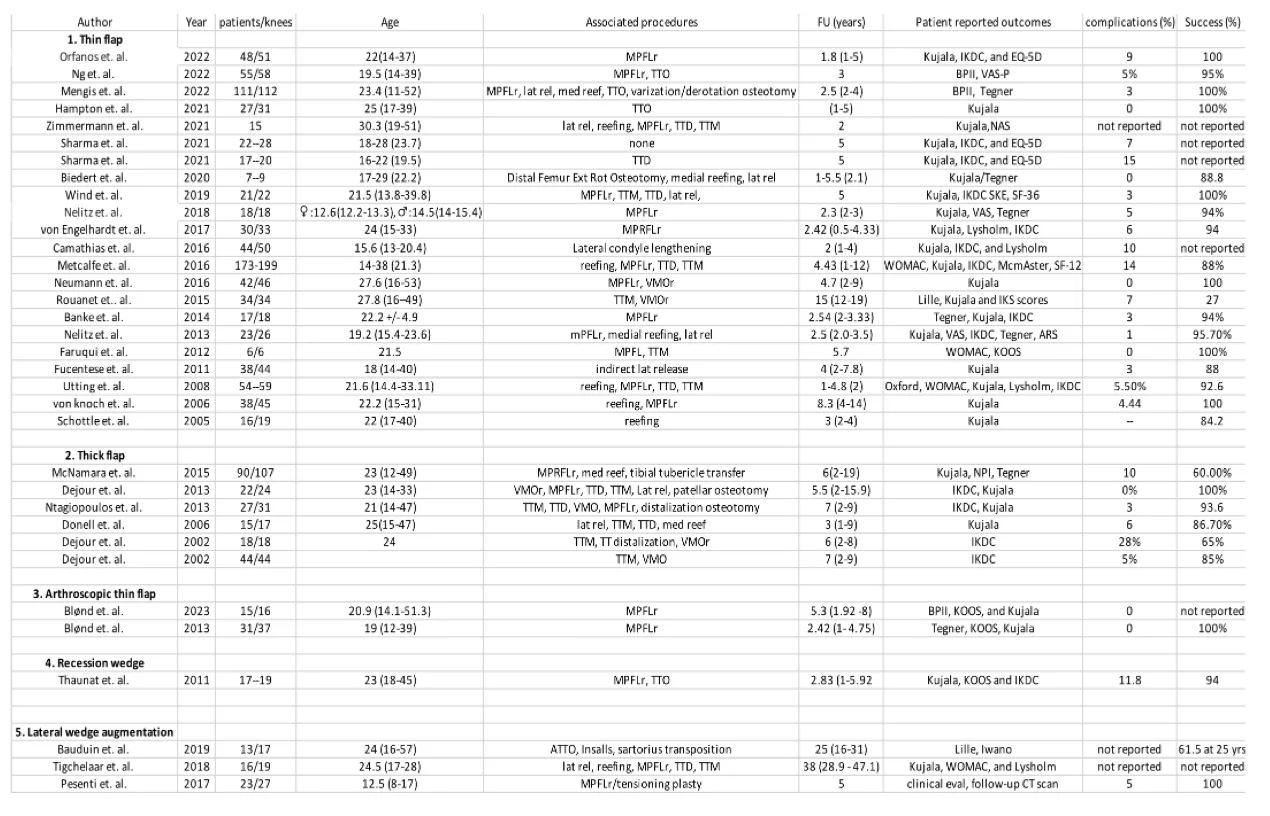
Trochleoplasty techniques including demographic features, associated procedures, outcome scores and complication rates.

A systematic review by Longo et. Al.^36^ which included 392 knees in 371 patients identified Bereiter deepening trochleoplasty as the most commonly performed technique with the lowest rates of recurrent instability, postoperative osteoarthritis and post-operative stiffness. However, the highest mean post-operative Kujala score was obtained by the Dejour procedure. The re-dislocation rate after the Bereiter, Dejour and Goutallier trochleoplasty were 0.8%, 3.2% and 10.5% respectively. This review concluded that none of the 3 commonly performed trochleoplasty techniques demonstrated significant superiority and all of them markedly enhanced stability and function, with a relatively low incidence of osteoarthritis and pain, and a moderate occurrence of complications.

Zimmermann et. al.^37^ published a case series of 15 patients with a minimum 2 year follow-up who underwent correction for patellofemoral malalignment with deepening trochleoplasty and concomitant realignment procedures. They noted considerable improvement in knee joint function and reduced pain while normalizing patellotrochlear congruence. In a case series of 111 patients with a 2–4-year follow-up, those who underwent thin flap trochleoplasty combined with medial patellofemoral ligament reconstruction or medial reefing, with or without accompanying realignment procedures, showed improvements in patient-reported outcomes and a high rate of return to sports. Two-thirds of the patients returned to their preoperative Tegner activity level or achieved a higher level. The probability of returning to the preoperative activity level (Tegner score) was significantly greater in the low-level activity group compared to high-level athletes.^38^

Dejour et al.^24^ retrospectively analyzed a series of 22 patients who underwent sulcus-deepening trochleoplasty between 1993 and 2006 for recurrent patellar instability after failed previous surgeries which showed satisfactory correction of patellar stability, improved radiological findings of patellofemoral instability, increase of functional scores and good patient satisfaction with no major complications. At a mean follow-up period of 66 months, 95.4 % of the patients had returned to their previous activities, including recreational sports with no recurrence of instability. All the patients responded that they were satisfied with the surgery. However, McNamara et. al.^39^ published that Dejour trochleoplasty does not result in a significant enhancement in sports participation at a competitive level. However, it does facilitate patient involvement in sports and exercise, particularly in non-twisting activities.

Similar to other minimally invasive surgical procedures, arthroscopically performed trochleopasty is advantageous over open procedure in terms of postoperative pain, infection, arthrofibrosis, postoperative scar and recovery period.^40^ In a case series of 16 knees in 15 patients undergoing Arthroscopic Deepening trochleoplasty and MPFLr with a mean follow-up of 63.6 months Blønd et. al.^41^ noted statistically significant and clinically meaningful improvements in patient-reported outcomes and standardized MRI measurements of trochlear dysplasia. These improvements were comparable to those achieved through open trochleoplasty, with no significant decrease in cartilage thickness.

Proximal trochleoplasty or Grooveplasty was a technique described by Peterson et. al.^42^ to reshape the proximal trochlea without altering the native distal trochlear groove. A recent study comparing Grooveplasty with trochleoplasty highlighted a higher activity level and higher degree of patella facet chondromalacia at baseline in the Grooveplasty cohort. At average follow-up period of 3.9 years, no recurrent instability was noted in the Grooveplasty group compared to 33% in trochleoplasty group. However, the functional outcome scores, complication rates and reoperation rates were similar in both groups of patients. Thus, Grooveplasty may be an alternative treatment option to full trochleoplasty in management of trochlear dysplasia.^43^

### Complications

The major complications after trochleoplasty surgery include worsened pain, postoperative stiffness and patellofemoral osteoarthritis. A systematic review of 14 studies examining the three most common trochleoplasty techniques revealed a relatively low complication rate, with an overall complication rate of 40%. These included pain, stiffness, and arthritis while the patella redislocation rate was 2%.^44^ However, the overall complication rate varies widely from 6.7% ^58^ to 13 %^7^ to 40%.^44^ The frequency of each complication rate varies widely in literature as shown in table below.

**Table 3.**
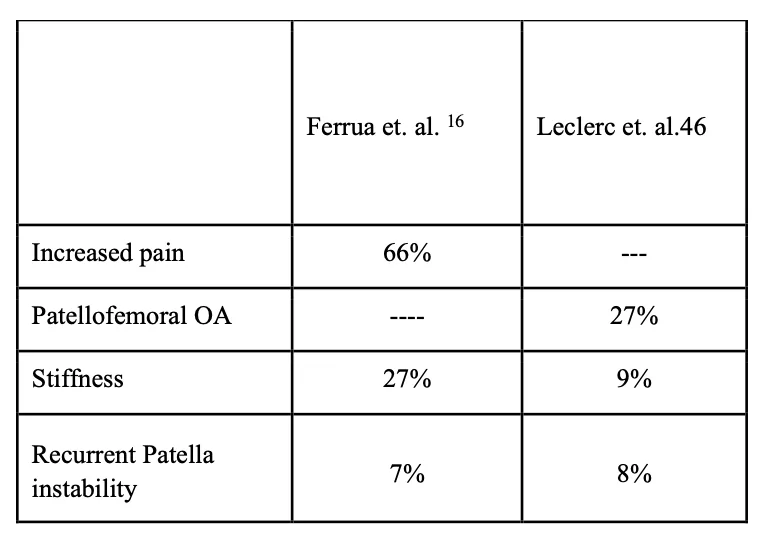
Frequency of each complication rate.

Pain is quite frequent after trochleoplasty as many patients have pre-existing chondral defects which increases the risk of residual pain and/or trochleoplasty is done after failure of previous surgery. Trochleoplasty is therefore not indicated for pain relief but to address instability. About the development of patellofemoral osteoarthritis following trochleoplasty, the current literature is inconclusive.^12,15,24,45^ Rounet et. al.^45^ reported a 97% incidence of patellofemoral osteoarthritis at 15-year follow-up after deepening trochleoplasty. Osteoarthritic changes were observed in about 18% of patients during long-term follow-up following Bereiter trochleoplasty.^44^

Schmeling^46^ reported that in a series of 488 open trochleoplasties over a 17-year period, none of the cases progressed to joint replacement, despite the fact that most patients had pre-existing cartilage damage at the time of the procedure, with some having significant cartilage damage (Outerbridge grade 3 to 4).

Arthrofibrosis is a potential complication following patellofemoral surgery and its incidence varies widely in literature. The varying findings on its occurrence, with Verdonk et al.^47^ reporting 46%, Donell et al.^48^ reporting 33%, and von Knoch et al.^12^ observing its complete absence in the long-term study, make it difficult to draw definitive conclusions. Early postoperative rehabilitation and emphasis on regaining motion reduces risk of postoperative stiffness. Additionally, if arthrofibrosis occurs, manipulation under anesthesia combined with lysis of adhesions has shown favorable results.

The average incidence of residual instability after various trochleoplasty techniques was 2%^36^ compared to 7% in isolated MPFLr in trochlea dysplasia patients.^12^ The recession type trochleoplasty had the highest rate (10.5%), followed by the Dejour (3.2%) and Bereiter (0.8%) techniques.^36^ Interestingly, all studies report no residual instability when a combination of Bereiter trochleoplasty with concurrent MPFL reconstruction.^40^

## CONCLUSION

Current literature demonstrates that thin flap sulcus deepening trochleoplasty is a viable procedure to manage patellofemoral instability in presence of severe trochlea dysplasia. It has been proven to have good clinical outcomes with acceptable complication rates. However, majority of the available evidence is case series studies. There is a need for higher level of comparative research to better define the indications and appropriate surgical technique as well as clarify long-term radiological and clinical functional outcomes.

Indeed, there is a lack of a comprehensive clinical and functional outcome measure which will help quantify the various aspects of patellofemoral joint. There is a dearth of robust evidence with regard to postoperative rehabilitation of trochleoplasty patients as well as return to sport criteria.

### Conflict of interest

None
